# Se nanoparticles stabilized with *Allamanda cathartica* L. flower extract inhibited phytopathogens and promoted mustard growth under salt stress

**DOI:** 10.1016/j.heliyon.2022.e09076

**Published:** 2022-03-08

**Authors:** Rajesh Dev Sarkar, Mohan Chandra Kalita

**Affiliations:** Department of Biotechnology, Gauhati University, Guwahati, Assam, India

**Keywords:** *Allamanda cathartica* L., Antibacterial, Mustard, Nanofertilizer, Selenium nanoparticle, Salt stress

## Abstract

Selenium Nanoparticles (SeNPs) exhibit tremendous application in agriculture as antimicrobials or as nano fertilizer. Present work reports the eco-friendly synthesis of SeNPs by using *Allamanda cathartica* L. flower extract (aqueous) as a reducing/capping agent and selenium dioxide as a precursor. The method used here is free of any toxic reducing agents and organic solvents. The synthesis process of SeNPs took 5 h at 60 °C, confirmed by the brick red colour of the solution followed by UV-Vis spectroscopy and further characterized by XRD, FTIR, EDX and SEM. The average size (diameter) of the SeNPs were found to be 60.31 nm by DLS. It has shown strong antimicrobial activity against *Pseudomonas marginalis* and *P. aeruginosa* at 2.5, 5 and 10 mg/mL concentrations. Besides, its application improved seed germination and growth parameters of *Brassica campestris* (TS 36 variety) under salt stress. 25 mg/L SeNPs has improved the germination percentage by around 31%, shoot length by 92%, root length by 78% and total chlorophyll content by 49% under 200 mM NaCl stress. This SeNPs could be a potential antimicrobial agent in treating plant diseases caused by the mentioned phytopathogens, having no or minimum toxicity, in fact having positive impacts on plant growth.

## Graphical abstract

**Figure:**
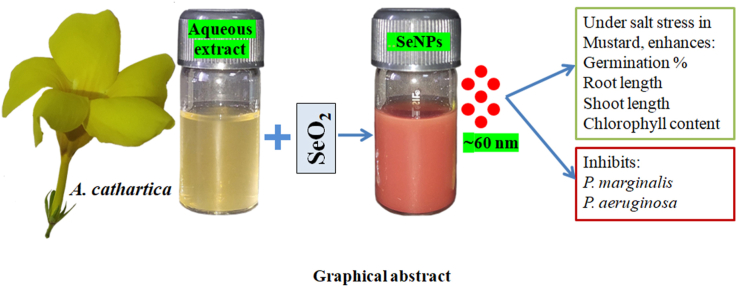

## 1 Introduction

Selenium Nanoparticles (SeNPs) have been proved to be an essential element in human as well as in lower plants such as algae, but in higher plants, though some beneficial activity has been observed still it is not proved to be essential (Araie and Shiraiwa 2009; El-Ramady et al., 2016; Sarkar et al., 2021a). At low concentrations (20–25 mg/L), SeNPs don't have any toxicity in higher plants, in fact, it promotes the growth and productivity of certain crops (Gudkov et al., 2020; Bano et al., 2021) and also alleviates environmental stress (Djanaguiraman et al., 2018; Morales-Espinoza et al., 2019; Soleymanzadeh et al., 2020; Shalaby et al., 2021). Other nanoparticles (NPs) such as SiO_2_ (Siddiqui et al., 2014), Ag (Almutairi, 2016), ZnO (Latef et al., 2017), TiO_2_ (Khan, 2016), CeO_2_ (Rossi et al., 2016), Fe_2_O_3_ (Askary et al., 2017), K_2_SO_4_ (El-Sharkawy et al., 2017), and Cu (Hernandez-Hernandez et al., 2018) also proved to protect various plants from salt stress-induced complications. When subjected to salt stress, high accumulation of reactive oxygen species (ROS) takes place in plants resulting oxidative stress (AbdElgawad et al., 2016). Selenium being incorporated into the amino acid selenocysteine regulates the activity of the antioxidant enzyme glutathione peroxidase, thereby playing a great role in protecting organisms from oxidative stress (Baker et al., 1993; Cartes et al., 2005). Besides SeNPs possess antimicrobial properties against many pathogens at certain concentrations (Alam et al., 2019; Fardsadegh and Jafarizadeh-Malmiri, 2019; Cittrarasu et al., 2021; Sarkar et al., 2021b) at the same time it possesses plant growth-promoting activity, which is a big advantage of treating fungal and bacterial plant diseases by SeNPs having no toxicity in crops. SeNPs have been green synthesized by many plant extracts (Sharma et al., 2014; Sasidharan et al., 2015; Satgurunathan et al., 2017; Alam et al., 2019; Fardsadegh and Jafarizadeh-Malmiri, 2019; Cittrarasu et al., 2021) by different researchers, but none focused on *A. cathartica* L. flower extract mediated green synthesis of SeNPs, which has great potential in agriculture. *A. cathartica* L. is traditionally well known throughout the world for its significant medicinal property as antimicrobial, antidiabetic, antihypertensive, anti-inflammatory and many more (Petricevich and Abarca-Vargas, 2019). Its flower contains quercetin (a strong reducing agent), kaempferol, hesperetin and some other flavonoid compounds (Ghosh et al., 2019) which may have a potential role in the synthesis of SeNPs and its stabilization. Phytopathogens such as *Pseudomonas aeruginosa* strains present in soil interact with plant roots and causes necrosis (Walker et al., 2004) and *Pseudomonas marginalis* causes severe stalk rot disease in mustard (Singh et al., 2016), *Fusarium oxysporum* (Williams and Saha 1993) *and Sclerotinia sclerotiorum* (Bharti et al., 2016) also causes severe harm to mustard cultivation. On the other hand, salt stress causes a significant effect on seed germination and growth of many crop plants including mustard (Shah, 2007; Benincasa et al., 2013; Zaman et al., 2015; Singh et al., 2019). Mustard is considered as one of the most important edible oil crops in some Asian countries including India and reduction in yield of this crop can cause major impact in the economy of the country. Salt stress causes a major harm to mustard crops, causing abnormality in osmosis and cell injury by excess ions entering the transpiration stream which finally obstructs seed germination, plant height, chlorophyll content and total seed yield affecting several metabolic pathways of plants (Singh et al., 2019). The present work mainly focuses on the optimized biosynthesis of SeNPs by using *A. cathartica* L. flower extract; evaluation of its potential in promoting *Brassica campestris* seed germination under salt stress and in inhibiting the growth of phytopathogenic microorganisms, especially pathogens that cause diseases of the mustard crop.

## 2 Materials and methods

### 2.1 Preparation of flower extract

**Figure 1:**
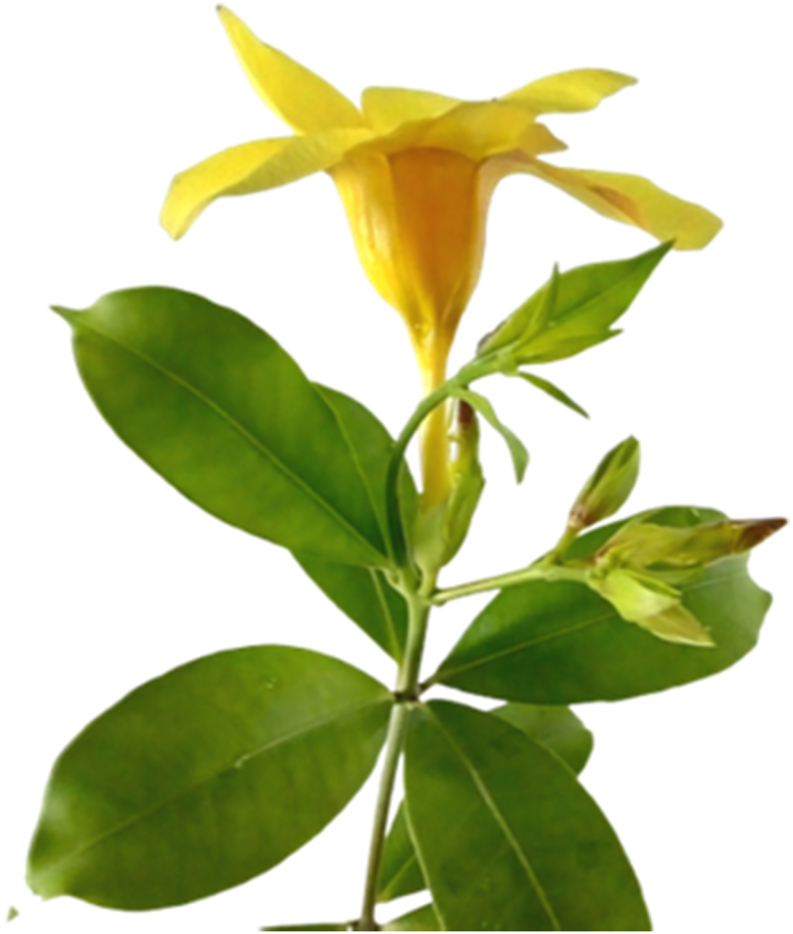
*Allamanda cathartica* L. twig with a flower.

Fresh *Allamanda cathartica* L. flowers (Figure 1) were collected from Gauhati University campus, Guwahati, Assam, India (latitude 26°09′02.35″N, longitude 91°39′25.36″E) and washed thoroughly by sodium hypochlorite once and by distilled water twice. Then 50 g of flower was crushed finely in a mixer grinder adding few mL of distilled water. It was then heated for 30 min at 80 °C, cooled and filtered through Whatman no. 1 filter paper. Centrifuged the filtrate at 7100 g for 10 min to remove unwanted organic matters. The supernatant was then collected, made volume up to 50 mL with distilled water and stored at 4 °C for further use. This extract is considered as 100% flower extract since 50 mL extract is made from 50g flower. Besides, for the purpose of proper identification, the herbarium of the plant was prepared and submitted to GUBH (Gauhati University Botanical Herbarium), Department of Botany, Gauhati University, Assam, India having accession no: GUBH19799.

### 2.2 Synthesis of SeNPs and its optimization

For the synthesis of SeNPs, *A. cathartica* L*.* flower extract was used as reducing/capping agent and SeO_2_ (brought from Merck) as the precursor molecule and the reaction was conducted at 60 °C at a continuous stirring condition in a magnetic stirrer. Optimization of the synthesis process was conducted by considering mainly four parameters- (a) concentration of the flower extract, (b) concentration of the precursor SeO_2_, (c) pH of the reaction mixture and (d) time required for completion of the reaction. The synthesis process was monitored by using a UV-Vis spectrophotometer. To optimize the concentration of flower extract; 10%, 15%, 20%, 25% and 30% flower extracts were used, keeping constant SeO_2_ concentration of 20 mM. For optimization of the precursor concentration 15 mM, 20 mM, 25mM, 30mM and 35mM SeO_2_ were used, keeping constant flower extract concentration of 20%. For optimization of the pH, reaction solution pH of 4, 6, 8, 10 and 12 were maintained by NaOH, keeping constant 20% flower extract and 25mM SeO_2_. Then the time required for the synthesis was optimized keeping the flower extract concentration at 20%, SeO_2_ concentration at 25mM, pH 10 and the reaction was monitored by taking UV-Vis spectra up to 6 h. After optimization of all four parameters, SeNPs has been synthesized in large amounts for its characterization and evaluation of its activity.

### 2.3 Extraction and purification of the SeNPs

After synthesis, the colloidal solution containing SeNPs was then centrifuged at 7100 g for 60 min. The pellet obtained was washed once with 70% ethanol followed by thrice with double distilled water by centrifugation at 20000 g for 20 min to remove the unwanted organic impurities and NaOH and finally, the pure SeNPs pellet was resuspended in 0.5 mL deionized water, ultra-sonicated for 10 min keeping in ice-cold water, poured in a clean glass slide and air-dried in a sterile chamber. These purified and fully dried SeNPs powder were stored at 4 °C for characterization and further experiment.

### 2.4 Characterization of the SeNPs

Synthesis of SeNPs was first detected by the formation of brick red coloured colloids in the reaction mixture, then scanning the solution by using a UV-Visible spectrophotometer (Thermo Scientific Multiscan Go) in the wavelength range of 250–700 nm. Fully dried purified SeNPs powder was used for the XRD (X-ray diffraction) analysis by X'Pert Pro Powder X-ray diffractometer operated at voltage 40 kV, current 40 mA with Cu-Kα radiation (K = 1.5436Å), for 2θ = 5°C–80 °C to determine the crystalline properties and purity of the green synthesized nanoparticles. SeNPs powder was ground with KBr to make pellet and analysed by FTIR Spectrophotometer (PerkinElmer Spectrum 2 Spectrometer) in the spectral range of 4000-400 cm^−1^ to determine the functional groups attached to the surface of the SeNPs. SeNPs were then coated with gold to determine the morphology and percentage purity by Scanning Electron Microscope (Sigma 300, Zeiss) coupled with Energy Dispersive X-ray Spectroscopy (EDAX-element). Nanoparticles were dispersed in distilled water and sonicated (by Hielscher ultrasonic processor, UP200Ht) to determine the particle size distribution and average particle size through DLS (Dynamic Light Scattering) technique by using Zeta Sizer (Malvern, Nano-s90).

### 2.5 Evaluation of the antimicrobial activity of the SeNPs

Plant pathogenic bacteria *Pseudomonas marginalis* (MTCC 2758) and *Pseudomonas aeruginosa* (MTCC 7925) were procured from MTCC, IMTech Chandigarh. SeNPs solution was prepared in three different concentrations viz., 2.5, 5 and 10 mg/mL in autoclaved distilled water and ultrasonicated for analysis of its antimicrobial property against *P. marginalis* and *P. aeruginosa*. Mueller-Hinton Agar (MHA) media plate was prepared (pouring equal volume of media in each plate) and 100 μL of bacterial suspension (maintained in McFarland standard using a spectrophotometer) was finely spread using a sterile glass spreader. Moreover, using a sterile micropipette tip five wells of 5 mm diameter were made in the MHA media, for agar well diffusion antimicrobial assay (Magaldi et al., 2004; Sarkar et al., 2022). SeNPs solution and Chloramphenicol (0.2 mg/mL) were loaded in the wells as mentioned in Table 1. Bacteria plates were then incubated at 37 ± 1 °C for 24 h. The experiment was done in triplicate and the zone of inhibition (Figure 8) was calculated as mean diameter ±SD (Standard Deviation), presented in Table 1.

**Table 1:** **Zone of inhibition by green synthesized SeNPs against phytopathogens:** Data presented here is the mean ± SD of 3 replicates for each strain of bacteria. The data were analyzed with one way ANOVA using the software Origin pro 8.5 and it was found that at 0.05 level the mean values are significantly different.

Well No.	Contents in well (50 μL in each well)	Zone of inhibition in mm (mean diameter ±SD)	
		*P. marginalis*	*P. aeruginosa*
1	Chloramphenicol (0.2 mg/mL)	20.33 ± 1.25	16.33 ± 1.25
2	SeNPs (2.5 mg/mL)	11.33 ± 1.25	10.67 ± 1.25
3	SeNPs (5 mg/mL)	19.00 ± 1.41	17.67 ± 1.25
4	SeNPs (10 mg/mL)	23.67 ± 1.25	22.00 ± 1.41
5	Flower extract (20%)	0	0

### 2.6 Assessment of the activity of SeNPs in seed germination and chlorophyll content of mustard (*Brassica campestris*) under salt stress

*Brassica campestris* L. (TS 36 variety) seeds were collected from Assam Seeds Corporation Limited (ASCL), Guwahati, India. Surface sterilized seeds were spread on Petri plates as shown in Figure 8 (25 seeds per plate) containing bloating paper soaked with Hoagland's solution (Hoagland and Arnon, 1950) containing SeNPs (12.5, 25 and 50 mg/L) and NaCl (200 mM) as presented in Table 2 and kept for germination in dark, at 18 °C. Plates containing Hoagland's solution was taken as control. For each treatment, three replicate plates were taken where each plate was loaded with 25 seeds. After 4 days, seed germination was analysed and the germination percentage of the seeds was calculated by Eq.(1).

(1) Germination percentage (GP %) = (Gf/n) × 100

where Gf- number of germinated seeds and n- number of seeds used in the test.

**Table 2:** **SeNPs activity in growth parameters of *B. campestris* under 200 mM salt stress:** Data presented here is the mean ± SD of three replicates for each treatment. The data were analyzed with one way ANOVA using Origin pro 8.5 and it was found that at 0.05 level the mean values are significantly different.

SeNPs (mg/L)	NaCl (mM)	GP (%) (mean ± SD)	Shoot length (mean ± SD)	Root length (mean ± SD)	Chl a	Chl b	Total Chl
					(mg/g tissue) as (mean ± SD)		
0	0	94.4 ± 4.56	2.74 ± 0.31	2.52 ± 0.18	0.793 ± 0.010	0.288 ± 0.019	1.081 ± 0.021
0	200	66.4 ± 5.37	1.33 ± 0.31	1.13 ± 0.29	0.417 ± 0.011	0.123 ± 0.013	0.540 ± 0.011
12.5	200	68.0 ± 2.83	1.54 ± 0.14	1.81 ± 0.33	0.429 ± 0.004	0.144 ± 0.016	0.574 ± 0.019
25	200	87.2 ± 3.35	2.56 ± 0.29	2.02 ± 0.29	0.606 ± 0.015	0.200 ± 0.008	0.806 ± 0.020
50	200	60.0 ± 2.83	1.22 ± 0.41	0.98 ± 0.05	0.402 ± 0.013	0.131 ± 0.023	0.533 ± 0.028

(GP: germination percentage, Chl: Chlorophyll).

Plates were then exposed to 16/8 h- light/dark condition in culture room, at 18 °C and Light intensity of 280 μmol m^−2^s^−1^. On the 7^th^ day, root and shoot length were measured for the germinated seeds (Table 2). Leaf chlorophyll content was then measured by Arnon's method (Arnon, 1949) taking 0.5 g of leaves from one plate and ground in 50 mL of 80% acetone, centrifuged for 20 min at 12000 g and recorded the OD (optical density) of the filtrate at 645 nm and 663 nm wavelength in a spectrophotometer (taking 80% acetone as blank). Repeated for all the treatments and replicates and the results were expressed as mean ± SD in Table 2. Chlorophyll content that is chlorophyll a, chlorophyll b, and total chlorophyll was determined using Eqs. (2), (3), and (4) respectively.

(2) $$\text{Chlorophyll ​a ​}\left(\text{mg ​per ​gm ​tissue}\right)=\left[12.7\left(\text{OD}663\right)–2.69\left(\text{OD}645\right)\right]\frac{\text{V}}{1000\text{ ​W}}$$

(3) $$\text{Chlorophyll ​b ​}\left(\text{mg ​per ​gm ​tissue}\right)=\left[22.9\left(\text{OD}645\right)–4.68\left(\text{OD}663\right)\right]\frac{\text{V}}{1000\text{ ​W}}$$

(4) $$\text{Total ​Chlorophyll ​}\left(\text{mg ​per ​gm ​tissue}\right)=\left[20.2\left(\text{OD}645\right)+8.02\left(\text{OD}663\right)\right]\frac{\text{V}}{1000\text{ ​W}}$$

where, W=Weight of sample taken (0.5 gm), V= Final volume of 80% acetone (50 mL).

### 2.7 Statistical interpretation

The data has been analyzed by calculating the mean and standard deviation. ANOVA test has been conducted by using Origin Pro 8.5 to understand the significance level of the data obtained.

## 3 Results and discussion

### 3.1 Optimization of SeNPs synthesis and characterization

**Figure 2:**
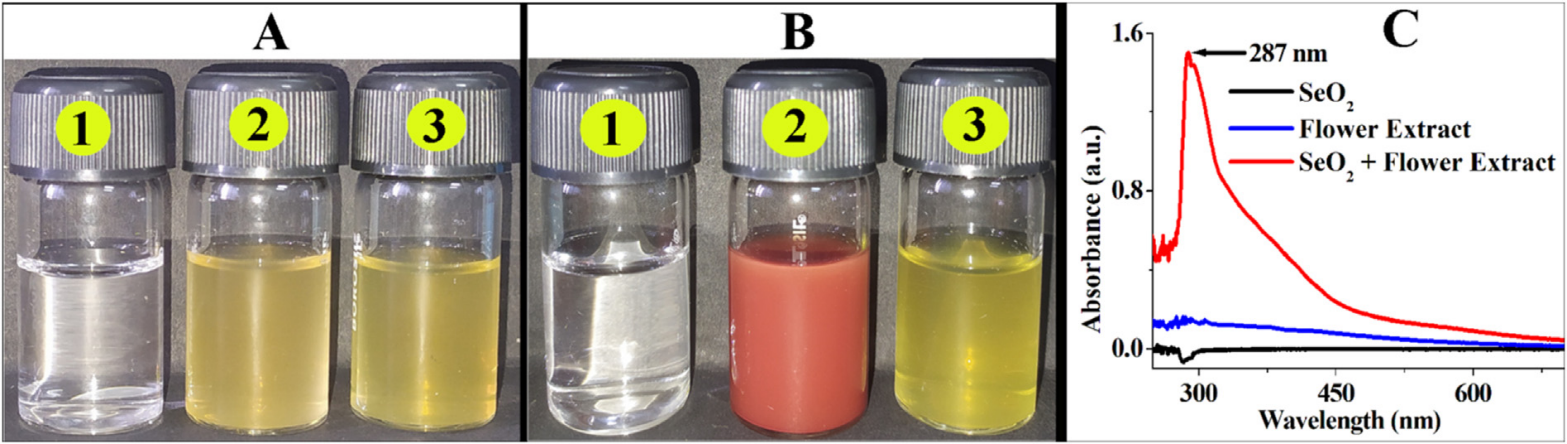
Synthesis of SeNPs by *A. cathartica* L flower extract (A) at 0 h (B) after 6 h (C) UV Vis spectra after synthesis, where (1) containing SeO_2_ solution, (2) SeO_2_ and flower extract and (3) flower extract only.

Synthesis of SeNPs by *A. cathartica* L. flower extract has been first detected by the colloidal brick-red colouration of the reaction solution (Figure 2A, Figure 2B) and then confirmed by UV absorbance peak of the synthesis solution (SeO_2_ + Flower extract) at the wavelength of 287 nm (Figure 2C), whereas there was no absorbance peak observed for the flower extract or the precursor SeO_2_ at that wavelength. The peak was formed due to the surface Plasmon resonance of the synthesized SeNPs. It took 5 h for the synthesis reaction to complete. Similarly, red SeNPs has been synthesized by Garlic extract (Satgurunathan et al., 2017), dried *Vitis vinifera* extract (Sharma et al., 2014), *Citrus reticulata* peel extract (Sasidharan et al., 2015) and *Glycosmis pentaphylla* leaf extract (Sarkar et al., 2022) from sodium selenite.

**Figure 3:**
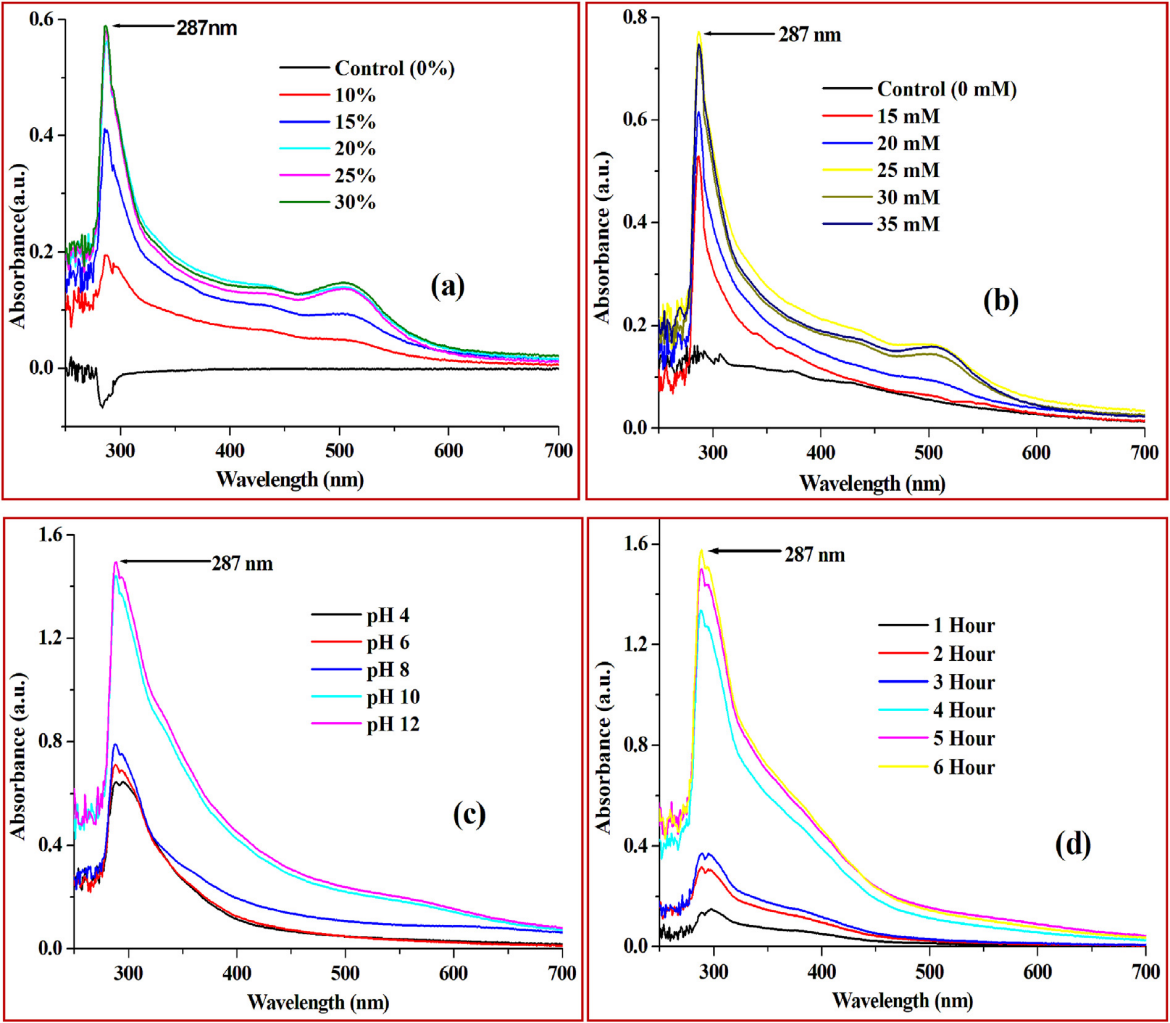
UV-Vis spectrum of SeNPs synthesized for optimization of (a) flower extract concentration, (b) SeO_2_ concentration, (c) pH of the solution and (d) time required for completion of the reaction.

In the process of optimization of flower extract concentration required for the synthesis of SeNPs from SeO_2_, keeping constant SeO_2_ concentration of 20 mM, it has been revealed that synthesis is increased while increasing flower extract concentration from 10% to 15% and 15%–20% but further increasing flower extract to 25% and 30% could not increase it significantly (Figure 3a). Hence 20% flower extract can be considered as the optimum concentration. Then taking constant flower extract concentration of 20%, the precursor (SeO_2_) concentration for the synthesis of SeNPs was optimized and found that SeNPs synthesis gradually increased by increasing SeO_2_ concentration up to 25 mM however beyond 25 mM, it doesn't show any significant increase of SeNPs synthesis (Figure 3b). Hence 25 mM is the optimum concentration of SeO_2_ for 20% flower extract for the synthesis of SeNPs. The synthesized SeNPs has shown an absorbance maximum peak at wavelength 287 nm. The two controls viz., SeO_2_ (Figure 3a) and flower extract (Figure 3b) did not show any peak at 287 nm. While optimizing the pH required for the reaction, it has been found that at pH of 4, 6 and 8 the synthesis is very low, however, it is suddenly increased to a great height at pH 10 compared to the lower pH. Further increasing pH to 12 did not increase the synthesis much (Figure 3c). Hence pH 10 can be considered as the optimum pH required for the synthesis process. The time required for completion of the synthesis process has been optimized and found that the synthesis keeps increasing up to 5 h at 60 °C and after that, there is no further significant increase hence the synthesis completes within 5 h at 60 °C (Figure 3d). Similarly, synthesis process of SeNPs by plant extracts have been optimized earlier for several parameters (Sasidharan et al., 2015; Alam et al., 2019).

**Figure 4:**
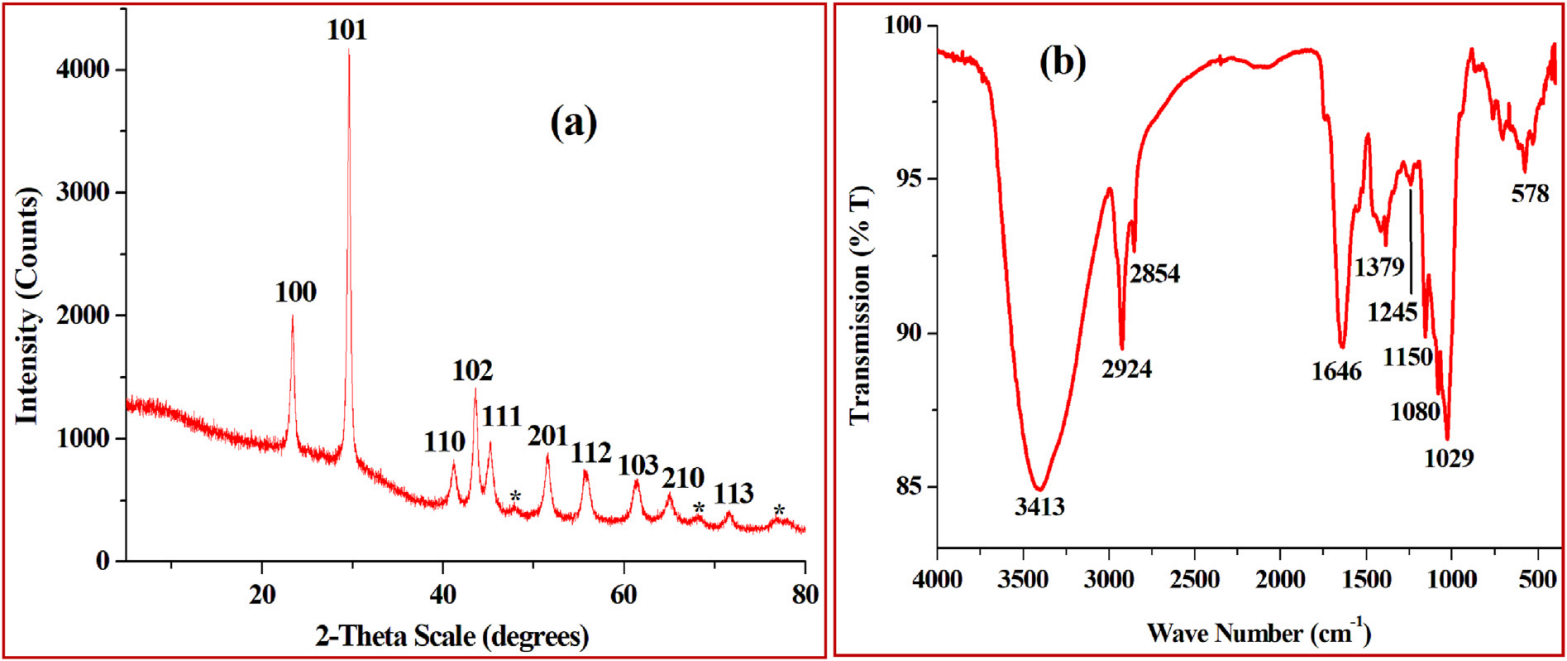
(a) XRD pattern and (b) FTIR spectra of SeNPs synthesized using *A. cathartica* L. Flower extract.

XRD data for the green synthesized SeNPs were analyzed and the diffraction peaks (Figure 4a) were observed at 2θ^°^ angles of 23.36^°^, 29.59^°^, 41.18^°^, 43.55^°^, 45.20^°^, 51.61^°^, 55.74^°^, 61.38^°^, 65.02^°^ and 71.47^°^ corresponding to lattice planes 100, 101, 110, 102, 111, 201, 112, 103, 210 and 113 respectively which are in good agreement with JCPDS (Joint Committee on Powder Diffraction Standards) data of SeNPs (JCPDS 06–0362) (Kumaran et al., 2011; Cittrarasu et al., 2021), clearly reveals the crystalline nature of the nanoparticles. Besides, three additional peaks (assigned as ∗ with 2θ^°^ angles of 47.99^°^, 68.09^°^, 77.06^°^) are present in the XRD data which appeared probably because of the presence of the phytochemicals of the flower extract.

To determine the functional groups on the surface of SeNPs, FTIR spectrum of SeNPs were recorded (Figure 4b) which showed significant absorbance bands at 3413 cm^−1^ corresponding to O–H stretch for alcohols and phenols groups (Cittrarasu et al., 2021), 2924 and 2854 cm^−1^ corresponds to C–H stretching of methylene groups of saturated aliphatic alkene, whereas 1646 and 1379 cm^−1^ represents C=C stretching of olefinic alkene and N–O stretching of aliphatic nitro compounds respectively (Satgurunathan et al., 2017; Nandiyanto et al., 2019). Peaks at 1245, 1150, 1080 and 1029 cm^−1^ can be attributed to the stretching of the carboxyl group (C=O) or carbonyl group (C–O) (Vyas and Rana, 2017). The FTIR analysis shows that several groups of compounds are involved in capping the synthesized SeNPs. These phytochemical compounds may also have role in exhibiting antibacterial activity and promoting seed germination of mustard.

**Figure 5:**
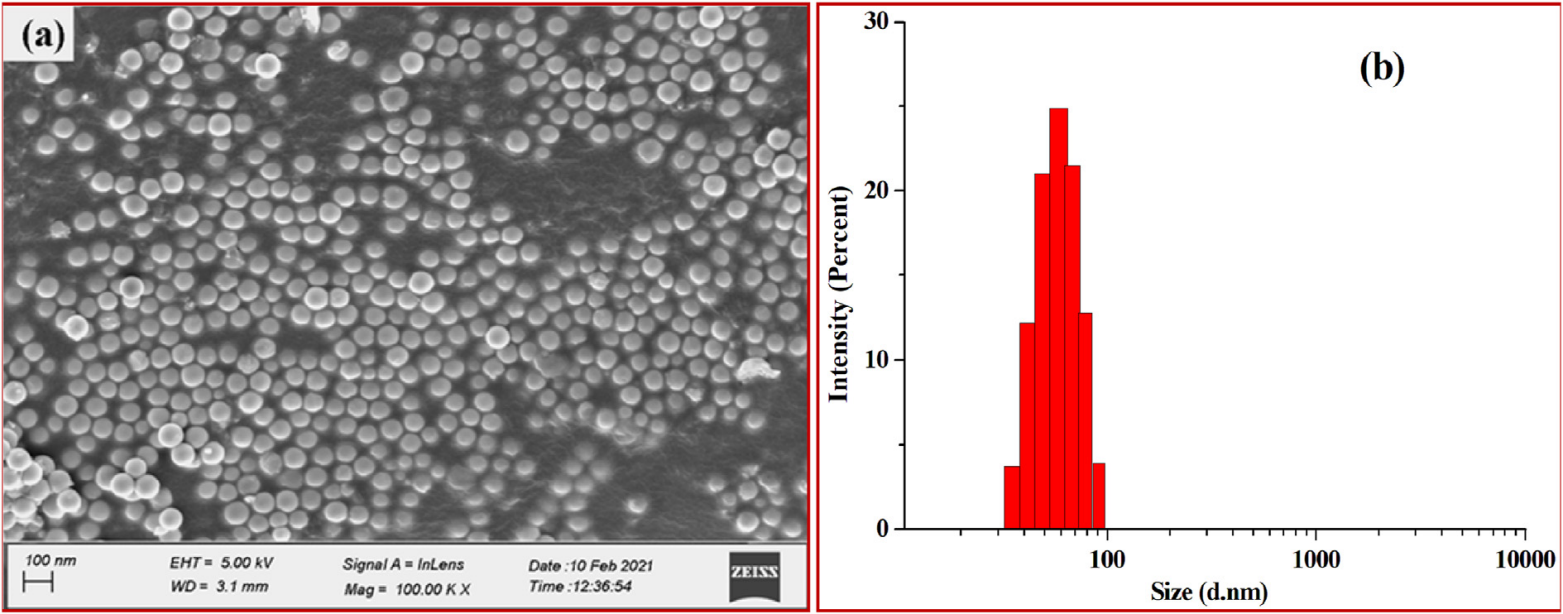
(a) SEM image and (b) DLS analysis of SeNPs.

**Figure 6:**
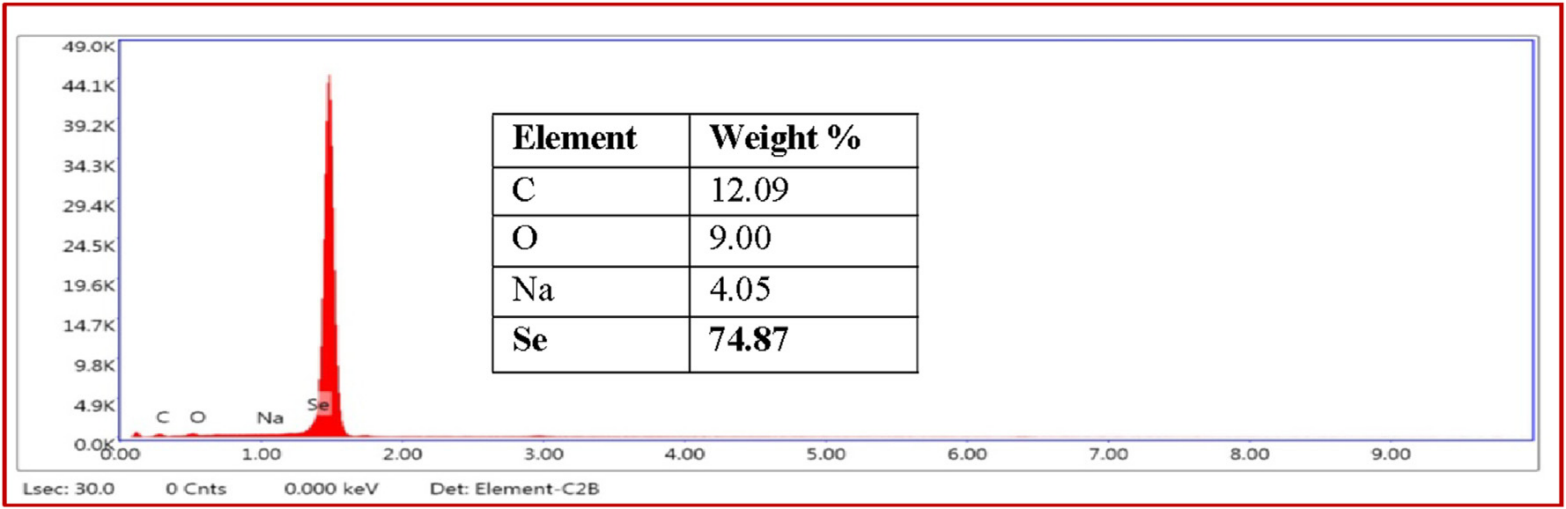
EDX spectra of SeNPs synthesized from SeO_2_ using *A. cathartica* L. Flower extract.

Morphological study of the green synthesized SeNPs was conducted by SEM (Sigma 300, Zeiss). The shape and size of the nanoparticles are the most important factor deciding their biological activity (Cheon et al., 2019). The SEM images revealed that SeNPs are mostly in spherical shape and the size range is below 100 nm (Figure 5a). Zeta-sizer revealed that the SeNPs are distributed in the size range of diameter 38–91 nm however most of the SeNPs (68%) in the solution were found to be in the range of 51–68 nm. Moreover, the average particle size (APS) of the SeNPs was found to be 60.31 nm diameters (Figure 5b). EDX spectra (Figure 6) of the green synthesized SeNPs showed that it contains 74.87 % Selenium, 12.09% Carbon, 9% Oxygen and 4.05% Sodium. Selenium being 74.87% is in quite a pure form and the carbon and oxygen present are due to the organic matters capping the SeNPs. The presence of Na is because of the addition of NaOH for maintaining pH.

### 3.2 Antimicrobial activity of the SeNPs

**Figure 7:**
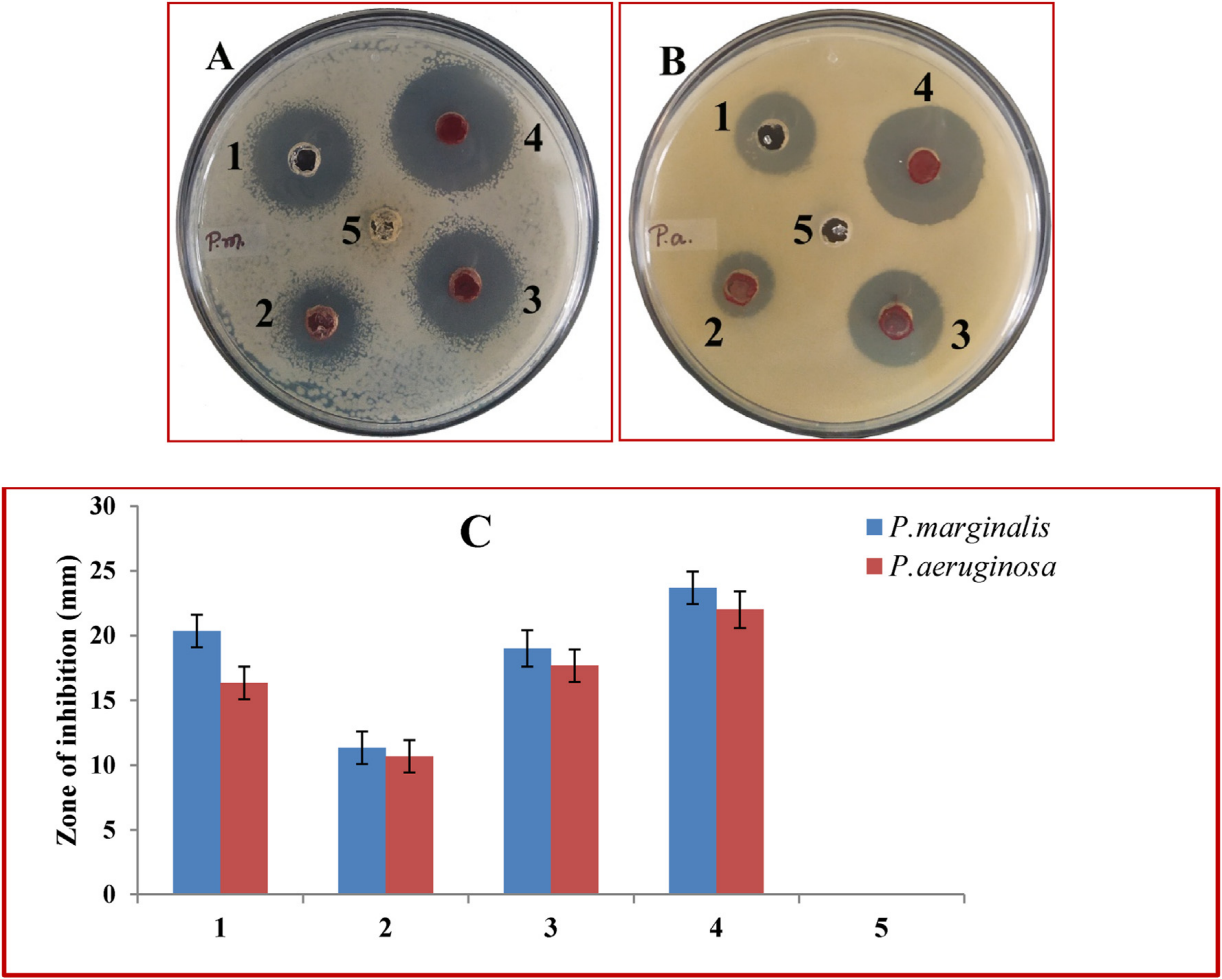
Antimicrobial activity of SeNPs against (A) *P. marginalis* and (B) *P. aeruginosa* by agar well diffusion assay. (C) Graph showing antimicrobial activity against A and B. [Here, well 1, 2, 3, 4 and 5 contains 50 μL of each of Chloramphenicol (0.2 mg/mL), SeNPs (2.5 mg/mL), SeNPs (5 mg/mL), SeNPs (10 mg/mL) and flower extract (20%) respectively]. The error bars in the graph represents the standard deviation.

The green synthesized SeNPs when tested for its antimicrobial activity at three different concentrations viz., 2.5, 5 and 10 mg/mL, against phytopathogenic bacteria, has shown a clear zone of inhibition circle in the range of 10.67–23.67 mm in *P. marginalis and P. aeruginosa* in agar well diffusion assay (Figure 7A, Figure 7B). The diameter of the zone of inhibition was compared with standard antibiotic chloramphenicol in Table 1. The antibacterial activity on both the bacterial strain has been compared (Figure 7C) and found that it has more activity against the bacteria *P. marginalis* than *P. aeruginosa*. However, the flower extract of *A. cathartica* didn't show any activity against these pathogens at that concentration of 20% flower extract. SeNP's antibacterial activity has been earlier tested for many bacteria including *P. aeruginosa* and other clinical pathogens (Sarkar et al., 2022), but phytopathogens like *P. marginalis* were never focussed for improvement in agriculture. Hence from the results of our study, it can be revealed that these low cost, tailored SeNPs can be implicated in agriculture for the treatment of these phytopathogens. Antibiotic activities of NPs are mostly due to selective blocking of bacterial enzyme activities by either competitive inhibition, non-competitive inhibition or denaturation of certain enzymes in a shape-dependent or size-dependent manner (Kopp et al., 2017). In a study ZnO NPs of three different shapes viz., pyramids, plates, and spheres with sizes below 20 nm inhibited the activity of *Escherichia coli* β-galactosidase enzyme (Cha et al., 2015). Moreover, SeNPs synthesized by *Ceropegia bulbosa* Roxb have successfully inhibited the growth of *Escherichia coli* and *Bacillus subtilis* in agar well diffusion assay (Cittrarasu et al., 2021). In some cases, the antibacterial activity of nanoparticles involves the formation of pits on the bacterial cell wall increasing permeability and causing cell death (Sondi and Salopek-Sondi, 2004). Hence our green synthesized SeNPs probably have undergone one of these mechanisms to inhibit the growth of the tested bacteria. Therefore, there is a great scope of this green synthesized SeNPs in agriculture sector to treat certain diseases in future, with further research mainly to understand the molecular mechanism of this activity.

### 3.3 Impacts of SeNPs on seed germination and chlorophyll content under salt stress

**Figure 8:**
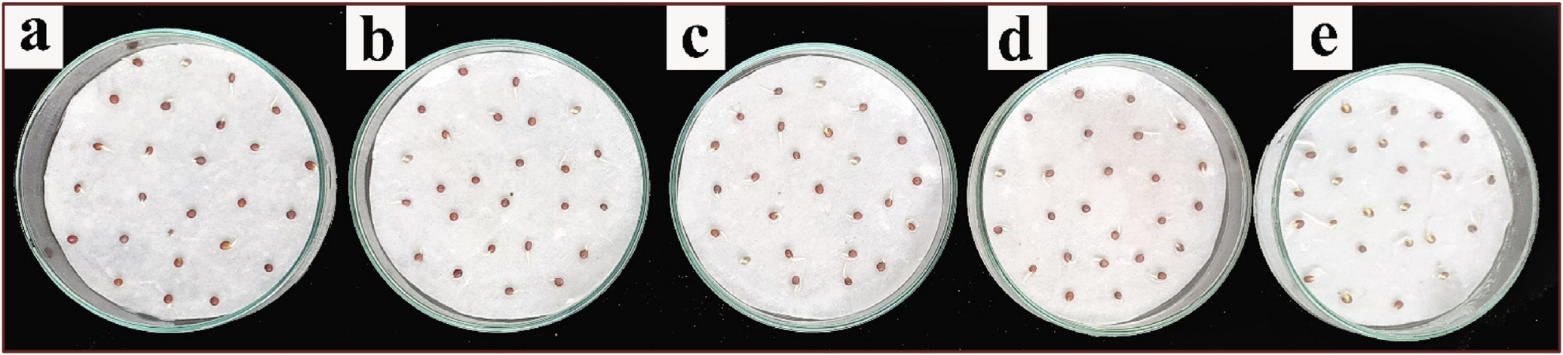
Seed germination test (a) 0 mg/L SeNPs +200 mM NaCl (b) 12.5 mg/L SeNPs +200 mM NaCl, (c) 25 mg/L SeNPs +200mM NaCl, (d) 50 mg/L SeNPs +200mM NaCl, (e) 0 mg/L SeNPs +0 mM NaCl.

**Figure 9:**
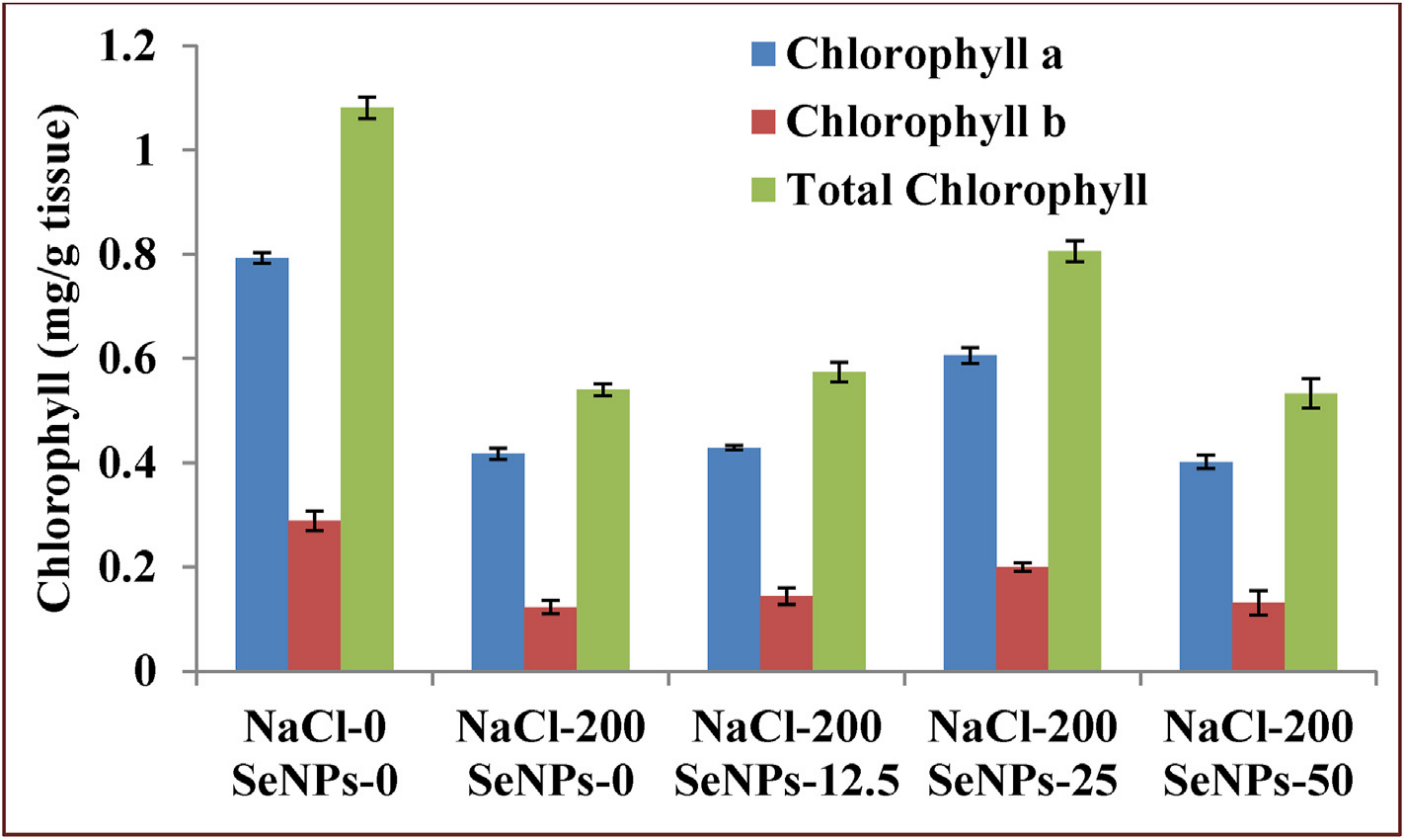
Impact of SeNPs in Chlorophyll content of *B. campestris* under salt stress. The error bars represent the standard deviation.

There has been a great reduction in seed germination percentage (Figure 8) and other growth parameters including root and shoot length, chlorophyll content (chlorophyll a, chlorophyll b and total chlorophyll) in *B. campestris* when subjected to salt stress of 200 mM NaCl. Green synthesized SeNPs application at concentrations of 12.5 and 25 mg/L in Hoagland's solution has improved the germination and growth parameters up to a significant height as presented in Table 2. The green synthesized SeNPs also played a great role in maintaining chlorophyll content (Chlorophyll a, Chlorophyll b and total chlorophyll) under salt stress condition (Figure 9). The chlorophyll content is a valuable deciding factor for the growth and production of any plant. In broccoli sprouts, 100 ppm nano selenium application has significantly enhanced the chlorophyll a content, however the chlorophyll b content was not affected (Vicas et al., 2019). Here in our work the best activity of SeNPs has been observed at 25 mg/L concentration that increased the GP% by around 31%, shoot length by 92%, root length by 78%, total chlorophyll content by 49%. However further increasing the SeNPs concentration to 50 mg/L caused a reduction in all the growth parameters, which implies that the concentration of 50 mg/L SeNPs is toxic to the seeds for its growth parameters under salt stress. In a recent study in *Brassica napus* L, SeNPs have significantly alleviated Cd toxicity by reducing the production of Cd induced reactive oxygen species by inhibiting the expression of oxidase enzymes *BnaRBOHC*, *BnaRBOHD1*, *BnaRBOHF1* and *BnaGLO* (Qi et al., 2021). Earlier SeNPs activity has been evaluated under salt stress in tomato (Morales-Espinoza et al., 2019), strawberry (Zahedi et al., 2019; Soleymanzadeh et al., 2020), *Brassica napus* (El-Badri et al., 2021) and cucumber (Shalaby et al., 2021) that improved several growth and physiological parameters including the activity of several antioxidant enzymes (catalase, superoxide dismutase, ascorbate peroxidase) thereby moving towards establishing a potential solution for crop cultivation in salt stress affected soil.

## 4 Conclusions

There exists a controversy of whether Se is an essential plant micronutrient or not because the study on the importance of Se in plants is still in a nascent phase. Though bulk Se is mostly toxic, the nano form of Se is proved to protect plants from environmental stress conditions including salt stress in some crops. Green synthesized SeNPs application in mustard under salt stress improved the basic growth parameters such as seed germination percentage, root and shoot length, chlorophyll a, chlorophyll b and total chlorophyll content as well as inhibited phytopathogenic bacteria in agar well diffusion assay. The current situation of global warming and many cyclones have salinized a huge cultivation area deteriorating the fields for cultivation of any crops, thereby downgrading the economy. Our findings on the importance of SeNPs in alleviating salt stress complications in oil crop mustard will develop alternate ways to cultivate the crop under salt stress affected areas. Simultaneously for treating several mustard crop diseases caused by *P. aeruginosa* or *P. marginalis* this green synthesized SeNPs can be utilized. Future investigation should be continued for elucidating the exact molecular mechanism of the activity of SeNPs in both inhibiting bacterial growth and promoting plant growth, and also focus should be given on technology advancement for large scale and low-cost production of SeNPs by green synthesis route for application in agriculture field.

## Declarations

### Author contribution statement

Rajesh Dev Sarkar: Conceived and designed the experiments; Performed the experiments; Analyzed and interpreted the data; Wrote the paper.

Mohan Chandra Kalita: Analyzed and interpreted the data; Contributed reagents, materials, analysis tools or data.

### Funding statement

Rajesh Dev Sarkar was supported by the Department of Science and Technology (DST), Government of India in the form of DST-INSPIRE Fellowship Program for PhD.

### Data availability statement

Data included in article/supplementary material/referenced in article.

### Declaration of interests statement

The authors declare no conflict of interest.

### Additional information

No additional information is available for this paper.
